# Reducing the actuation threshold by incorporating a nonliquid crystal chain into a liquid crystal elastomer

**DOI:** 10.1039/c7ra11165g

**Published:** 2018-01-29

**Authors:** Hongyan Niu, Yuchang Wang, Jun Wang, Wenlong Yang, Yinmao Dong, Meng Bi, Jindi Zhang, Jiaojiao Xu, Shuyue Bi, Binsong Wang, Yachen Gao, Chensha Li, Jianqi Zhang

**Affiliations:** Key Laboratory of Chemical Engineering Process and Technology for High-Efficiency Conversion, School of Chemistry and Material Sciences, Heilongjiang University Harbin 150080 P. R China 2005037@hlju.edu.cn; Key Laboratory of Functional Inorganic Material Chemistry, Ministry of Education of the People's Republic of China, Heilongjiang University Harbin 150080 P. R China lichnsa@163.com lichensha@hlju.edu.cn; Key Laboratory of Electronics Engineering, College of Heilongjiang Province, Heilongjiang University Harbin 150080 P. R China gaoyachen@hlju.edu.cn; Department of Applied Science, Harbin University of Science and Technology Harbin 150080 P. R China; School of Sciences/Beijing Key Lab of Plant Resource Research and Development, Beijing Technology and Business University Beijing 100048 China; College of Medicine Information Technology, Heilongjiang University of Chinese Medicine Harbin 150040 P. R China; Key Laboratory of Nanosystem and Hierarchical Fabrication, National Center for Nanoscience and Technology Beijing 100190 P. R. China zhangjq@nanoctr.cn

## Abstract

Liquid crystal elastomers (LCEs) are important smart materials that can undergo reversible deformation in response to liquid crystal (LC) phase transitions. A low threshold temperature for LC phase transition is advantageous because the LCE material can be more conveniently actuated by the applied stimulus. In this work, we investigated the effect of a nonliquid crystal chain on the reduction of threshold temperature of the LC phase transition by linking a nonliquid crystal side chain, 4-methoxyphenyl-1-hexenyloxy (MOCH_3_), to the network backbone of a classical polysiloxane-based side-chain nematic LCE. The nematic–isotropic transition temperature (*T*_ni_) of the MOCH_3_ incorporated nematic LCE was lower than that of the normal nematic LCE without the incorporation of a nonliquid crystal chain by about 27 °C. Compared to the normal nematic LCE or its nanocomposite, the MOCH_3_ incorporated nematic LCE or its nanocomposite demonstrated more rapid thermo-actuated deformation or photo-actuated deformation, and can be actuated to attain full axial contraction at an obviously lowered temperature or by light with obviously lowered intensity, while the maximum contraction ratio basically did not vary. These research results indicate that some nonliquid crystal chains show potential for improving the characteristics and enhancing the application significance of LCE materials.

## Abstract 2

The incorporation of nonliquid crystal chains made the actuation threshold of LCE being obviously decreased, and the LCE material can be effectively actuated by a lower energy intensity of the applied stimulus.

## Graphical abstract

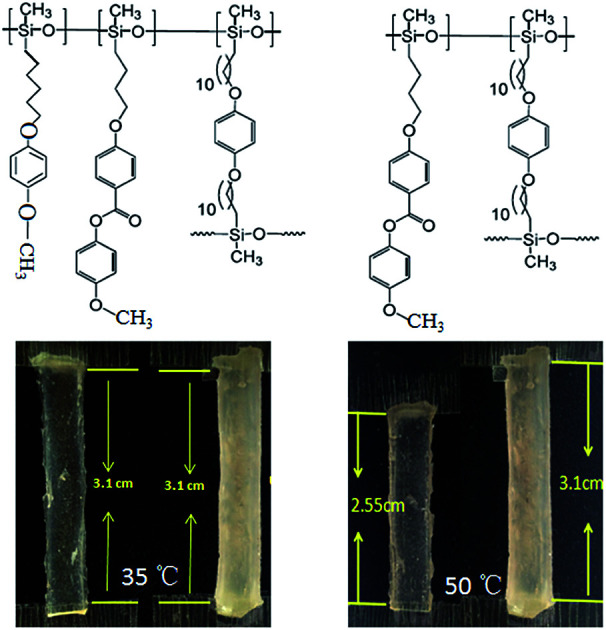

## 1. Introduction

As a unique type of smart polymer deformable materials, liquid crystal elastomers (LCEs) have been intensively studied in recent years due to their fascinating properties and potential applications.^1–6^ LCEs are mesogen-containing networks formed by lightly crosslinked polymer chains, which show both the anisotropic orientation characteristic of liquid crystals (LCs) and the rubbery elasticity of polymer networks, and endow the materials with specific properties, such as stimuli-induced reversible deformation and anisotropic shape changes. A typical LCE usually consists of three basic components: the mesogens, the polymer backbone and the crosslinkers. The mesogens connected to the polymeric backbone are the key element needed for formation of the liquid crystal phase. The crosslinkers covalently connect different polymer chains to build a network. This unique structure is the reason for the interaction between the LC order of the mesogenic units and the entropic elasticity of the rubber network. In the nematic state of LCE, the polymer chains adopt an anisotropic conformation induced by the coupling between backbone and mesogenic order. In the isotropic state, this coupling disappears and the polymer chains recover a random coil conformation driven by entropy.^7,8^ At the nematic–isotropic phase transition, such a change in the average macromolecular conformation, between the anisotropic conformation and the random coil conformation, will induce a reversible macroscopic shape deformation of a LCE sample. Therefore, LCE materials can possess several important features, such as large deformation, reversible actuation, relative ease to manipulate and control, *etc.*,^2,5,8^ which enable them to be potential in many applications, such as smart actuators,^9,10^ sensors,^11,12^ artificial organs,^13,14^ smart surfaces,^15–17^ and microrobots,^18,19^*etc.*

Most LCE materials are designed to be responsive by using heat or light stimuli. The thermal-responsive LCEs perform reversible deformations related to the temperature-determined nematic–isotropic phase transition,^20^ while the deformations of light-responsive LCE materials depend on either the molecular structure changes of the incorporated chromophores or the photo-thermal effect induced nematic–isotropic phase transition.^5,21–25^ One typical type of light-responsive LCE materials takes advantage of the photo-thermal effect of thermal conductive fillers to transform photon energy into heat which further induce the nematic–isotropic phase transition and thus actuate the LCE materials to deform.^10,17,18,26–32^ The nematic–isotropic phase transition always occurs at a specific temperature, which is the threshold temperature to produce the full actuation of a LCE material. In most cases, a low nematic–isotropic transition temperature (*T*_ni_) is desired, best slightly above ambient conditions, as this is energy efficient and facilitates the applicability. The *T*_ni_ can be influenced by several factors, such as the crosslinking density,^33^ the chemical structures of the mesogenic molecules and the network.^34–36^ Some researchers' work indicated that the other possible ways to reduce the *T*_ni_ should be by mixing two or more mesogenic molecules, or mixing the mesogenic molecules and nonliquid crystal molecules within the LCE network.^37,38^

Polysiloxane LCEs, distinguishing themselves from other LCE materials with the characteristics of much lower glass transition temperature, viscosities, surface energy, good mechanical and thermal stabilities, convenient fabrication process, and environment friendly behavior,^20^ are the most widely researched type of LCEs. A classical polysiloxane side-chain LCE, based on the hydrosilylation of polyhydrogen methylsilxoane (PMHS) with 4-methoxy-1-buteneoxy phenyl (MBB) as mesogen and 1,4-alkeneoxybenzene (11UB) as crosslinker, has been originally developed by Finkelmann and co-workers,^39^ and extensively studied in characteristics and explored in application over the years.^10,12,26,27,29,31,40–58^ In this work, we investigated the effect of nonliquid crystal chain on the reduction of *T*_ni_ by linking a nonliquid crystal side chain, 4-methoxyphenyl-1-hexenyloxy (MOCH_3_), to the network backbone of this polysiloxane side-chain LCE. Based on the hydrosilylation of PMHS with MBB, MOCH_3_ and 11UB, the synthesized network of the modified LCE contained mesogens and nonliquid crystal side chains linked to the polymer backbone. Experiment results exhibited that the *T*_ni_ of the modified nematic LCE was lower than that of the normal nematic LCE (without the nonliquid crystal side chains in its network) with the same crosslinking density by about 27 °C. Compared to the normal nematic LCE or its nanocomposite which was fabricated by filling graphene into the LCE matrix, the modified nematic LCE or its graphene filled nanocomposite demonstrated more rapid thermo-actuated deformation or photo-actuated deformation, and can be actuated to attain the full deformation (axial contraction) at an obviously lowered temperature or by the light with obviously lowered intensity, while the maximum contraction ratio basically did not vary. These results indicated that the addition of nonliquid crystal chain effectively improved the LCE characteristic, and the application potentiality of this classical nematic LCE material will also be greatly extended.

## 2. Experimental procedures

### 2.1 Materials preparation

The mesogen monomer: MBB, nonliquid crystal chain: MOCH_3_, and cross-linker: 11UB, were synthesized following literature reports.^10,21,26^ The polymer backbone was a poly-dimethylhydrosiloxane (PMHS) with approximately 60 Si–H units per chain, obtained from ACROS Chemicals (Belgium, USA). The commercial platinum catalyst, dichloro(1,5-cyclooctadiene) platinum(ii) (Pt(COD)Cl_2_), was obtained from Aldrich (St Louis, USA). The catalyst solution was prepared by dissolving 0.025 g of the dichloro(1,5-cyclooctadiene) platinum(ii) in 2 mL of dichloromethane, then adding 20 mL of toluene. The graphene was obtained from Xianfeng Nano-Tech (Nanjing, China).

**Scheme 1:**
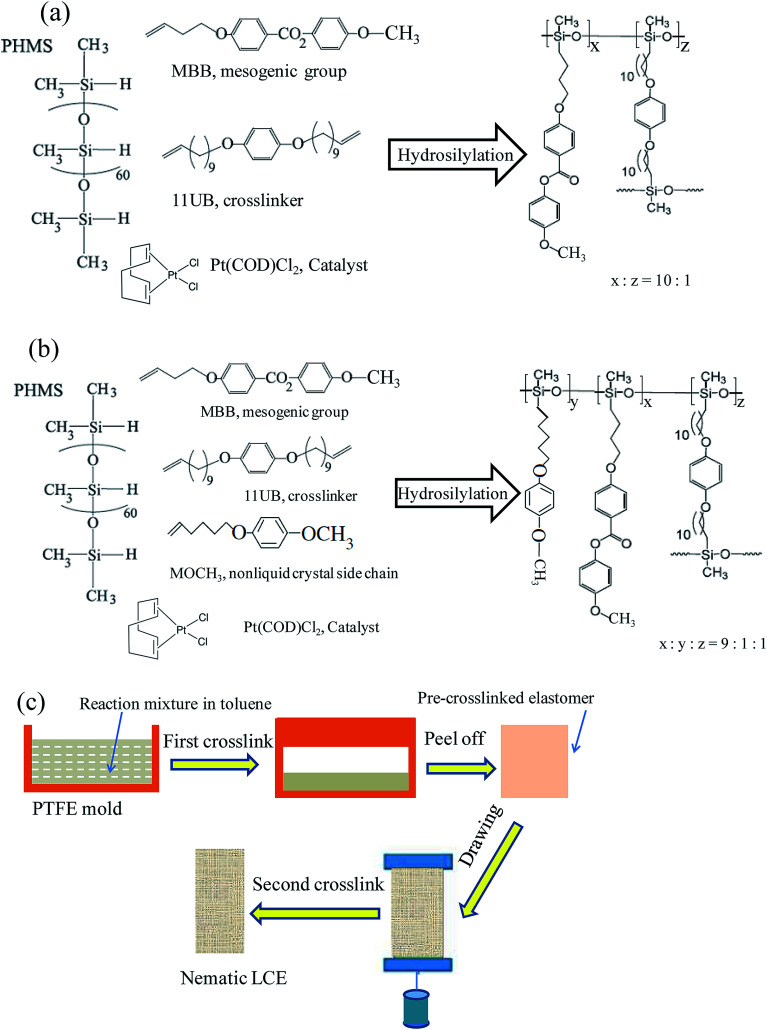
(a) The molecular structure of every composition of the reaction system for the synthesis of the normal LCE, and the network structure of the synthesized normal LCE. (b) The molecular structure of every composition for the synthesis of the modified LCE, and the network structure of the synthesized modified LCE. (c) Illustration of the preparation protocol of LCEs.

Scheme 1(a) and (b) illustrate the reaction systems for the syntheses of the normal LCE and the modified LCE, and the network structures of these two LCEs. The synthesis of the side-chain nematic LCE network with the polysiloxane backbone was *via* a sol–gel method, coupled with a two-stage crosslinking and a drawing process. The Si–H bonds in the PMHS backbone reacted with the terminal vinyl groups of the side pendent monomers (mesogenic molecules or nonliquid crystal chains) and the crosslinkers in the presence of the platinic acid catalyst for hydrosilation, achieving an effective 16% cross-linking density.

The preparation protocol of nematic LCEs is shown in Scheme 1(c). The reaction mixture for preparing the normal nematic LCE was 0.048 g PHMS, 0.2 g MBB (0.672 mmol), 0.0272 g 11UB (0.066 mmol) and proper amount catalyst solved in 0.8 mL of toluene. The reaction mixture for preparing the modified nematic LCE was 0.048 g PHMS, 0.18 g MBB (0.605 mmol), 0.0138 g MOCH_3_ (0.0672 mmol), 0.0272 g 11UB (0.066 mmol) and proper amount catalyst solved in 0.8 mL of toluene, the molar ratio between MBB and MOCH_3_ was 9 : 1. As shown in Scheme 1(c), the reaction mixture was cast into a Teflon (PTFE) rectangular parallel-piped mold with the dimensions of 4 cm × 1.2 cm × 1 cm. The mold was first ultrasonicated for 3 minutes to remove tiny bubbles entrained in the mixture, then put in an oven in 63 °C for 40 min for partial crosslinking process (first crosslinking stage). A toluene contained swollen gel of partially crosslinked elastomer was generated during this first crosslinking stage. Thereafter the mold was cooled to the room temperature. 4 mL of hexane was poured into the mold to facilitate the separation of the partially crosslinked elastomer from the mold. The swollen elastomer was carefully transferred out of the mold and dried for 40 minutes. As the contained toluene gradually evaporated, the elastomer shrunk to a stable size of 2.8 cm in length. The de-swollen elastomer was slowly uniaxially stretched under a load of 6 g weight for 12 hours to attain a stable length of about 4 cm (drawing process) and form the nematic phase in matrix network. Thereafter the elastomer with the load was heated at 70 °C overnight to complete the crosslinking reaction in nematic phase (second crosslinking stage) to obtain the normal nematic LCE or the modified nematic LCE. The LCE nanocomposites with the graphene as fillers (G–LCE) were prepared by the same procedure but adding 0.0008 g graphene in the reaction mixtures, the prepared normal nematic G–LCE and modified nematic G–LCE respectively contained the graphene of 0.3 wt% content.

### 2.2 Characterization methods

The LCE mesomorphic properties were observed using polarizing optical microscopy (POM, Nikon Instruments, SMZ 1500, Melville, NY). The two dimensional X-ray scattering (2D-WAXS) experiments were performed by a Bruker/Siemens Hi-Star 2D X-ray Diffractometer with a monochromatic CuKalpha point source (0.8 mm). The phase transformation was investigated by differential scanning calorimetry (DSC) measurements (TA Instruments Q100 modulated differential scanning calorimeter, New Castle, DE) at a heating and cooling rate of 10 K min^−1^. The thermo-actuation of the LCEs was performed by using a thermostat cabinet, and the photo-actuation of the LCE nanocomposites was performed by using a wide-spectrum light source (New Port, Oriel Sol3A, Irvine, CA). A universal material mechanical analyzer (CMT-10, LG Company, Jinan, China) was employed to measure the change of strain of the LCE materials under heating or irradiation with a pair of tension clamps along the material's stretch direction under a pre-applied stress of 60 kPa. The luminance intensity was measured by using an illumination instrument (FLUKE 941, Avery De, USA). The environment temperature of experiments was 20 °C.

## 3. Results and discussions

**Fig. 1:**
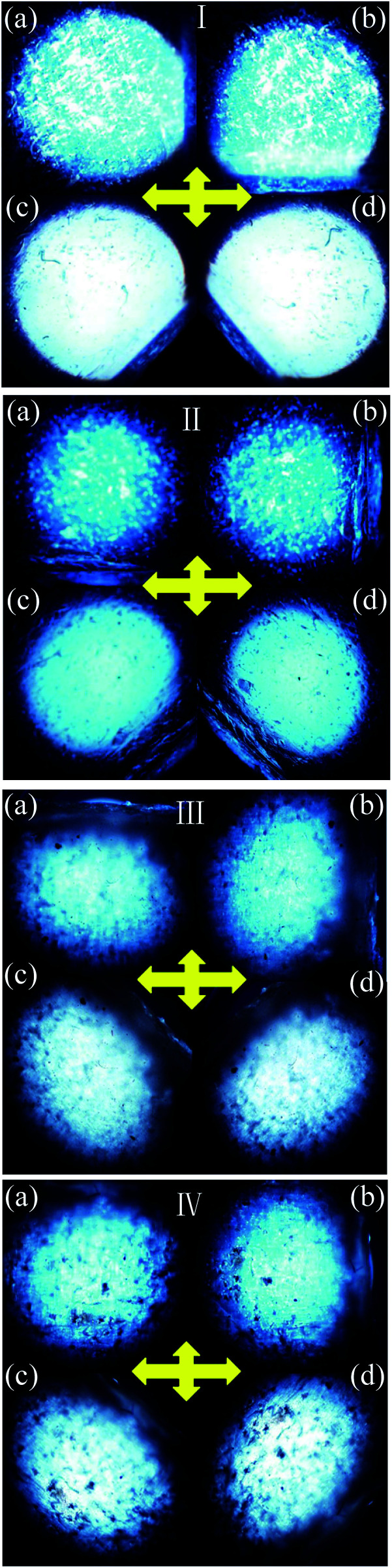
POMs of the normal nematic LCE (I), modified nematic LCE (II), normal nematic G–LCE (III) and modified nematic G–LCE (IV). Inserted cross arrows illustrate the polarization directions of the two polarizers. (a) The stretch direction of the LCE material is parallel to the vertical polarization direction. (b) The stretch direction of the LCE material is parallel to the horizontal polarization direction. (c) The angle between the stretch direction of the LCE material and the polarization direction of either polarizer is −45°. (d) The angle between the stretch direction of the LCE material and the polarization direction of either polarizer is 45°.

**Fig. 2:**
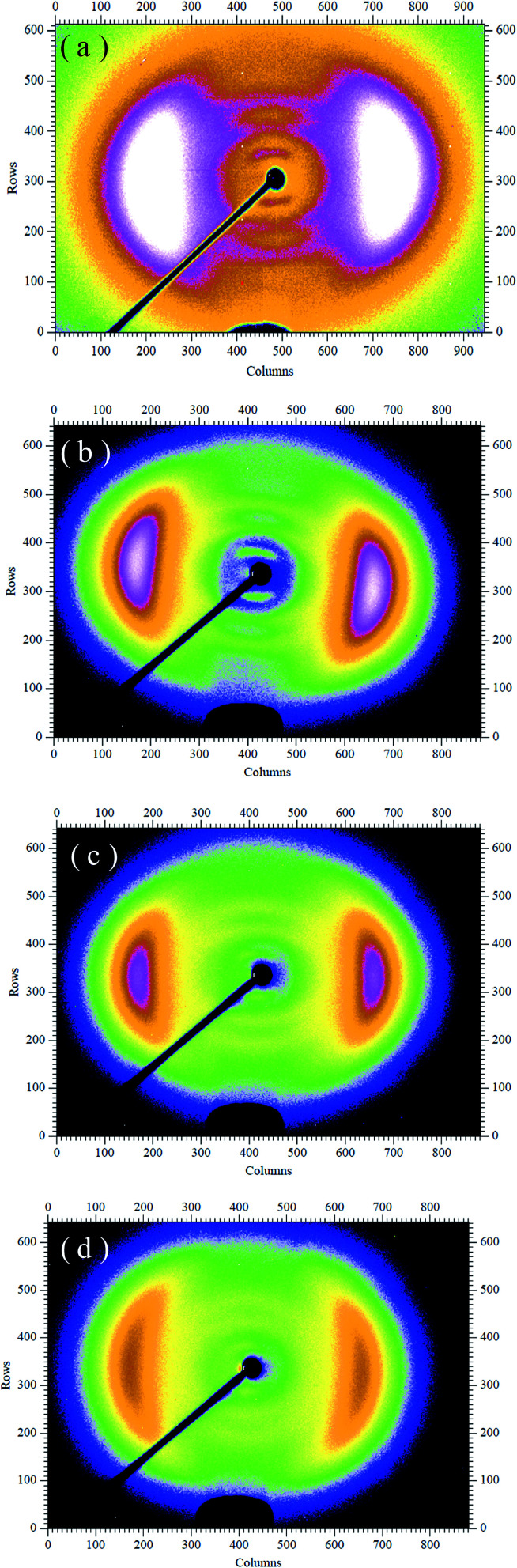
2D-WAXS patterns of the normal nematic LCE (a), modified nematic LCE (b), normal nematic G–LCE (c) and modified nematic G–LCE (d).

The POM was used to evaluate the uniaxial alignment effect of the mesogens in LCE matrix by measuring the transmittance of a probe light through two crossed polarizers with a LCE material sample between them. As shown in Fig. 1, the highest transmittance appeared when the angle between the stretch direction of the LCE material and the polarization direction of either polarizer was ±45°, while the lowest appeared when the stretch direction was parallel to one of the polarization directions. Periodic changes of dark and bright images were observed by rotating the sample with an interval of 45°. The POM observations of the normal LCE, modified LCE, normal G–LCE and the modified G–LCE exhibited consistent result, though due to the blocking of light by graphene, the transmittances in the images of the G–LCEs were relatively darker. The POM observations proved an LC nematic-phase structure, and that the mesogenic units were well aligned along the stretch direction. Fig. 2 shows the 2D-WAXS patterns of the LCE materials measured with the incident beam perpendicular to the material surfaces, the azimuthal intensity maxima at wide-angle reflections indicate alignments of mesogens in LCE matrices.^10,58^ The 2D-WAXS patterns of the four LCE materials also exhibited consistent result that the locations of the wide-angle reflection are orthogonal to the stretch directions of the materials, thus further confirmed the LC nematic-phase structure of LCE matrices, and that the mesogenic units were well aligned along the stretch direction. This nematic structure was attributed to the mechanic-induced molecular alignment effect. A partially crosslinked network was generated during the first crosslinking stage. When a uniaxial stress was applied to such a partially crosslinked network in the drawing process, the alignment state was established with the director along the stress axis, and was eventually fixed by the following second cross-linking stage, resulted in the nematic-phase structure in LCE matrix.

**Fig. 3:**
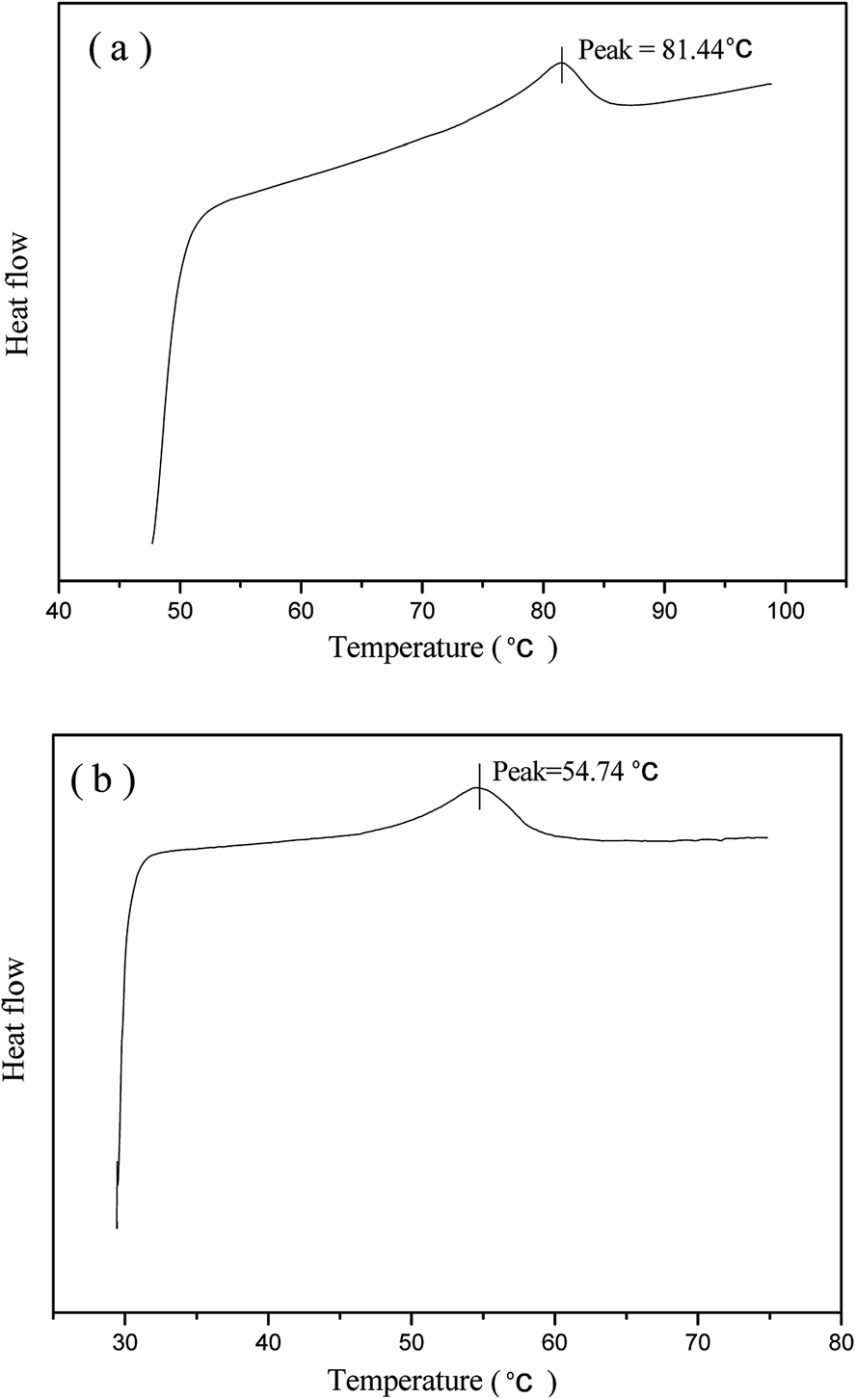
DSC data curves of the normal nematic LCE (a) and the modified nematic LCE (b).

Fig. 3 presents the DSC measurement of phase transformations in the prepared normal LCE and modified LCE. The *T*_ni_ measured from the DSC heating cycle of the normal LCE is 81.44 °C, while the *T*_ni_ of the modified LCE is 54.74 °C, lower than that of the normal LCE by about 27 °C. This result indicates that the incorporation of nonliquid crystal chain can play an important role in reducing the *T*_ni_ of LCEs. The network of the modified nematic LCE contained the mesogenic units and nonliquid crystal chains linked to the backbone as side pendant groups. The flexibility of nonliquid crystal chains effectively reduced the hindrance and weakened the packing interaction between the rigid mesogenic units, led to a lowered energy threshold for triggering the nematic–isotropic phase transition. The phase transition kinetic of LCE network is also determined by the Arrhenius equation *k* = *A* exp(−*E*_a_/*RT*),^34,59^ where *k* is the kinetic constant for phase transition, *A* is the pre-exponential factor, *E*_a_ is the activation energy of phase transition, *R* is the universal gas constant, and *T* is the temperature. It can be known from the Arrhenius equation that a higher temperature is needed to attain the LC phase transition for higher activation energy, while a lower temperature can realize the LC phase transition for lower activation energy. Just because the flexible nonliquid crystal chains influenced the anisotropic environment of the mesogenic units, led to the decrease of activation energy of phase transition, the *T*_ni_ of LCEs was obviously lowered.

**Fig. 4:**
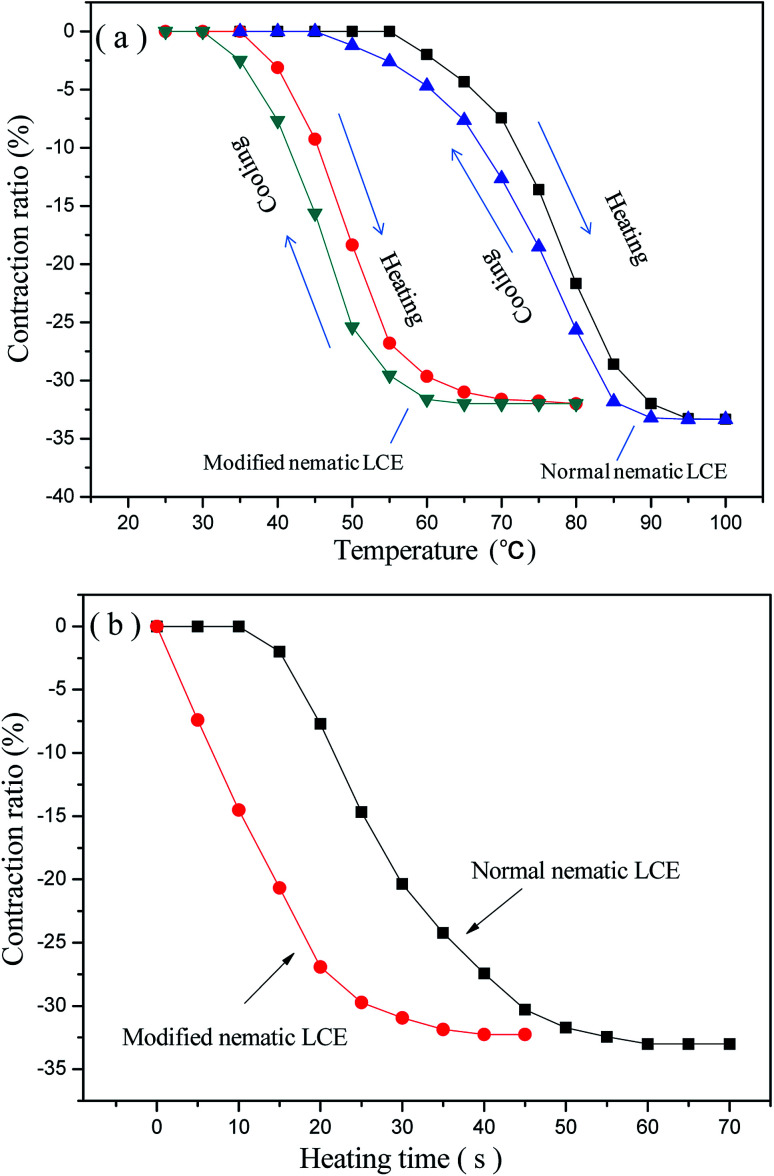
(a) The axial contraction ratios of the normal nematic LCE and the modified nematic LCE at different temperature; (b) heating time *versus* the axial contraction ratios of the normal nematic LCE and the modified nematic LCE under the heating temperature of 90 °C.

The prepared nematic LCE materials demonstrated thermo-actuation properties. They contracted along the alignment directions when heated, and regained their original lengths after cooling. The maximum axial contraction was about one-third of their original lengths. Fully reversible contraction and restoration was demonstrated under these heating/cooling cycles. The axial contraction ratios, which are the ratios between the contraction lengths and the original lengths, of the normal nematic LCE and the modified nematic LCE with the changing temperature are presented in Fig. 4(a). It can be seen that the thermo-actuated deformation mainly occurs near the *T*_ni_ of the LCEs. This deformation was induced by the change of the nematic order of the LCE matrix. When the nematic LCE matrix is heated above or cooled below its *T*_ni_, the change in the degree of alignment of mesogenic units changes the nematic order corresponding to a switch between the nematic structure and the isotropic state, results in spontaneous contraction or elongation of the matrix network along the alignment direction. As shown in Fig. 4(a), compared to the normal nematic LCE, the temperatures for the modified nematic LCE to attain the same axial contraction ratios obviously decrease due to the lower *T*_ni_. The temperature for the modified nematic LCE to attain the full axial contraction is lower than that for the normal nematic LCE by about 20 °C, while their maximum contraction ratios are basically equal. During the cooling processes back to room temperature, the axial contraction ratios gradually decrease to zero in a relaxation manner, revealing a full recovery of the original lengths of the LCEs. Our experiment also demonstrated that the contraction ratios of the two LCEs were basically constant contraction ratios after several tens of times of repeatable heating/cooling processes. The axial contraction ratios of the normal nematic LCE and the modified nematic LCE *versus* heating time under the temperature of 90 °C are plotted in Fig. 4(b). The contraction ratios gradually increased as the heating time being prolonged, and finally tended to be stable because the maximum contraction ratios were reached. The modified nematic LCE demonstrated more rapid contraction deformation relative to the normal nematic LCE, it attained its maximum contraction ratio at about 35 s while the normal nematic LCE attained its maximum contraction ratio at about 60 s. The calculated rates of contraction ratio of the modified nematic LCE and the normal nematic LCE from Fig. 4(b) were about 0.91% per s and 0.55% per s respective, and the calculated energy densities of contraction actuation of them were about 0.0472 J g^−1^ and 0.0461 J g^−1^ respective.

**Fig. 5:**
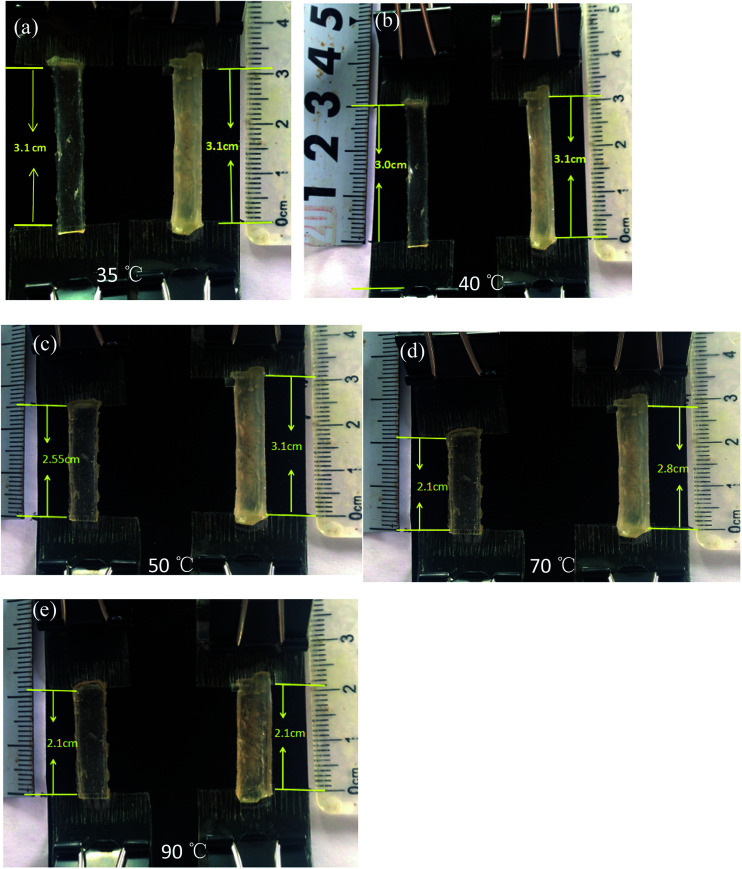
Photo images of the thermo-actuation of the modified nematic LCE (the left sample) and the normal nematic LCE (the right sample) at different temperature. Every sample has an initial length of 3.1 cm and is loaded by 3 g of weight during the thermo-actuation process.

Fig. 5 shows a series of intuitionistic comparison of thermo-actuation properties of the normal nematic LCE and the modified nematic LCE. The two LCE samples were hung on a horizontal stick inside the thermostat cabinet, and each of them was loaded by 3 g of weight attached at the bottom. Fig. 5(a) shows the two samples had a same initial length of 3.1 cm. Fig. 5(b) shows that the modified nematic LCE began to perform contraction behavior at the temperature of 40 °C which is far below its *T*_ni_, while the normal nematic LCE remained stationary. At the temperature of 50 °C which is near the *T*_ni_ of the modified nematic LCE, the normal nematic LCE still remained stationary while the modified nematic LCE performed a obviously contraction, contracted to the length of 2.55 cm, as shown in Fig. 5(c). At the temperature of 70 °C which is between the *T*_ni_ of the modified nematic LCE and the normal nematic LCE, the modified nematic LCE already reached to its maximum contraction, contracted to the final length of 2.1 cm, while the normal nematic LCE only performed a small amplitude of contraction, contracted to the length of 2.8 cm, as shown in Fig. 5(d). At the temperature of 90 °C which is above the *T*_ni_ of the two nematic LCEs, the two samples all contracted to their final length of 2.1 cm, as shown in Fig. 5(e).

**Fig. 6:**
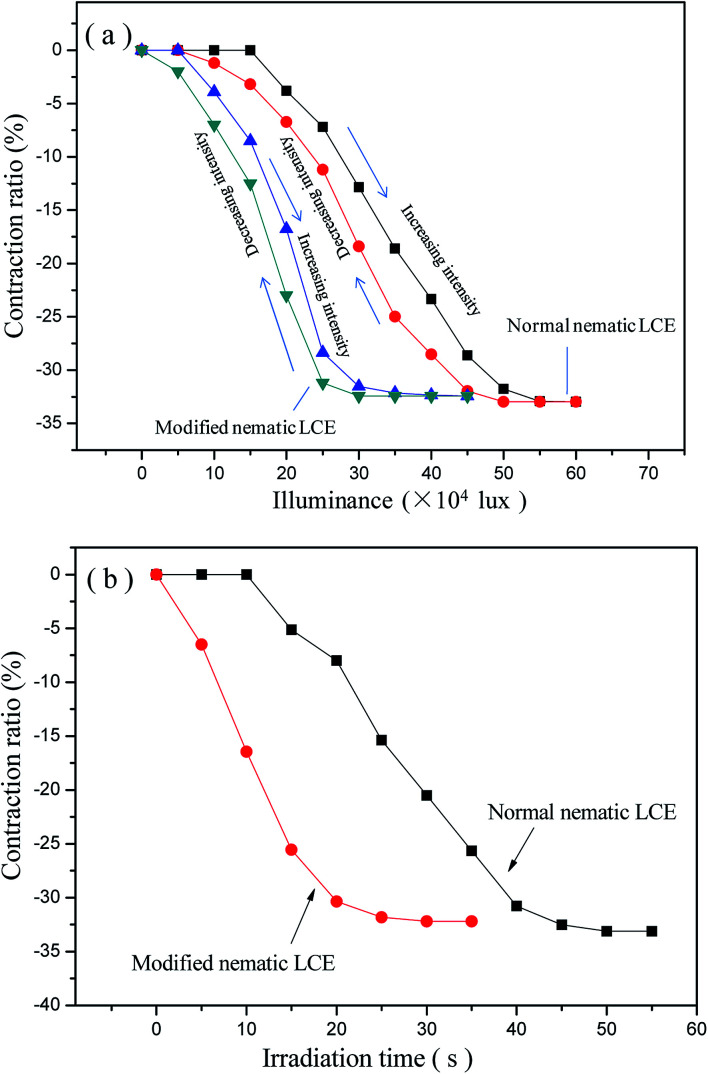
(a) The axial contraction ratio of the normal nematic G–LCE and the modified nematic G–LCE at different luminance intensity; (b) irradiation time *versus* the axial contraction ratios of the normal nematic LCE and the modified nematic LCE under the luminance intensity of 6.0 × 10^5^ lux.

Our experiments indicated that the photo-actuation properties of LCE materials were also obviously improved due to the reduction of *T*_ni_. In order to realize the photo-actuation, the G–LCEs were prepared by filling the graphene in LCE matrices, as described in “Experimental procedures” section. Both the normal nematic G–LCE and the modified nematic G–LCE performed reversible photo-actuated deformation behaviors, they contracted along the alignment directions under the irradiation of the used wide-spectrum light source, and regained their original lengths after the light source was removed. Fully reversible contraction and restoration was demonstrated under the cycles of irradiation and removing irradiation. The photo-actuation properties of the G–LCEs were ascribed to the effect of photo-thermal energy conversion of the graphene filled in the LCE matrices. Graphene can efficiently absorb and convert photo energy into thermal energy, thus acting as nanoscale heaters embedded in the LCE matrices. The absorbed thermal energy increased the temperature in the matrices, leading to the nematic–isotropic phase transition and deformation of the LCE nanocomposites. The normal nematic LCE and the modified nematic LCE were essentially photo-transparent with very low photo-thermal energy conversion efficiency, did not demonstrate photo-actuation behavior. The axial contraction ratios of the normal nematic G–LCE and the modified nematic G–LCE *versus* luminance intensity are plotted in Fig. 6(a), it indicates that the achieved axial contraction ratios under irradiation increase as the increase of luminance intensity until reaching to the maximum axial contraction ratios which are consistent with the values under thermo-actuation. This is because the higher irradiation intensity elevated the rate of photo-thermal energy conversion, and thus elevated the equilibrium temperature in LCE matrix, resulted in larger contraction amplitude. When the maximum contraction ratio was achieved, the matrix was in fully isotropic state and the material did not continue to contract as the increase of luminance intensity. Fig. 6(a) shows that compared to the normal nematic G–LCE, the modified nematic G–LCE began to perform photo-actuation deformation under a very lower luminance intensity, and also needed very lower luminance intensities to attain the same axial contraction ratios due to its lower actuation threshold. The luminance intensity for the modified nematic G–LCE to attain the full axial contraction is lower than that for the normal nematic G–LCE by about 2 × 10^5^ lux, which is about two times of the intensity of maximum natural sunlight, while their maximum contraction ratios are basically equal. During the processes of gradual decreasing the luminance intensity back to zero, the axial contraction ratios gradually decrease to zero in a relaxation manner, revealing a full recovery of the original lengths of the G–LCEs. Our experiment also demonstrated that the contraction ratios of the two G–LCEs were basically constant after several tens of times of repeatable irradiation and removing irradiation. The axial contraction ratios of the normal nematic G–LCE and the modified nematic G–LCE *versus* irradiation time under the luminance intensity of 6.0 × 10^5^ lux are plotted in Fig. 6(b). The contraction ratios gradually increased as the irradiation time being prolonged, and finally tended to be stable because the maximum contraction ratios were reached. The modified nematic G–LCE demonstrated more rapid contraction deformation relative to the normal nematic G–LCE, it attained its maximum contraction ratio at about 25 s while the normal nematic G–LCE attained its maximum contraction ratio at about 45 s. The calculated rates of contraction ratio of the modified nematic G–LCE and the normal nematic G–LCE from Fig. 6(b) were about 1.28% per s and 0.73% per s respective, and the calculated energy densities of contraction actuation of them were about 0.0495 J g^−1^ and 0.0484 J g^−1^ respective.

**Fig. 7:**
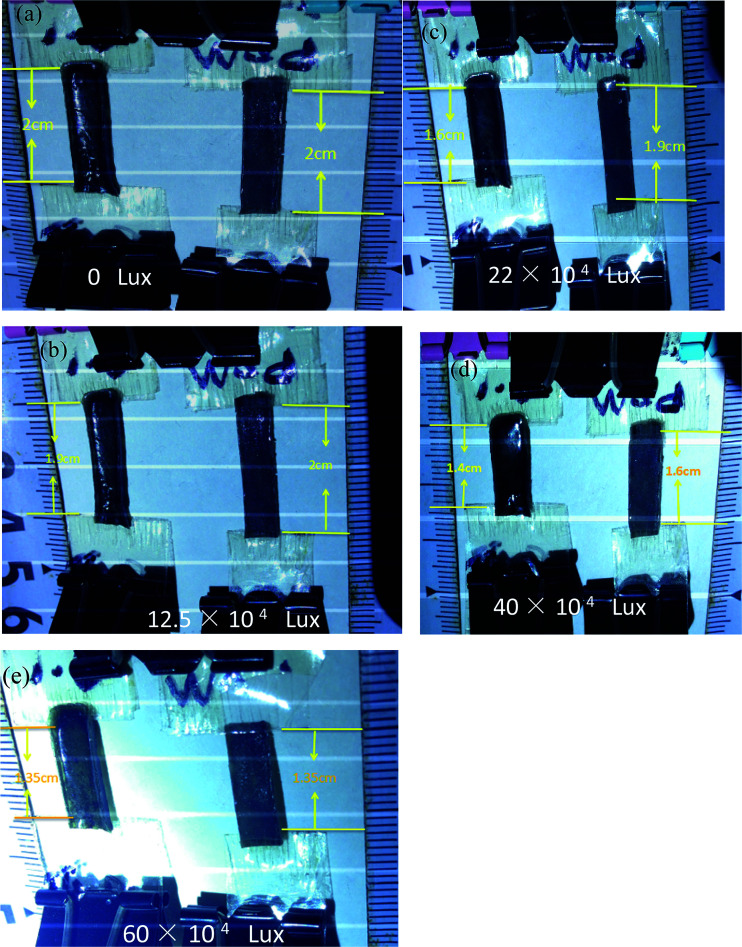
Photo images of the photo-actuation of the modified nematic G–LCE (the left sample) and the normal nematic G–LCE (the right sample) under different luminance intensity. Every sample has an initial length of 2.0 cm and is loaded by 3 g of weight during the photo-actuation process.

Fig. 7 shows a series of intuitionistic comparison of photo-actuation properties of the normal nematic G–LCE and the modified nematic G–LCE. The two G–LCE samples were hung on a horizontal stick, and each of them was loaded by 3 g of weight attached at the bottom. Fig. 7(a) shows the two samples had a same initial length of 2.0 cm. Under the irradiation of the used wide-spectrum light source, the modified nematic G–LCE began to perform contraction behavior under the luminance intensity of 1.25 × 10^5^ lux, while the normal nematic G–LCE remained stationary, as shown in Fig. 7(b). Under the luminance intensity of 2.2 × 10^5^ lux, the normal nematic G–LCE performed a small contraction of 0.1 cm while the modified nematic G–LCE performed a larger contraction, contracted to the length of 1.6 cm, as shown in Fig. 7(c). Under the luminance intensity of 4.0 × 10^5^ lux, the normal nematic G–LCE contracted to the length of 1.6 cm while the modified nematic G–LCE nearly reached to its maximum contraction, contracted to the length of 1.4 cm, as shown in Fig. 7(d). Under the luminance intensity of 6.0 × 10^5^ lux, the two samples all contracted to their final length of 1.35 cm.

## 4. Conclusions

The study of LCEs with low *T*_ni_ is an important way to enhance their applicability as actuator materials as they can be more conveniently actuated by the applied stimuli due to the low actuation thresholds. In this work, we investigated the effect of nonliquid crystal chains on reducing the *T*_ni_ of LCEs by linking a nonliquid crystal side chain, MOCH_3_, to the network backbone of a classical polysiloxane side-chain LCE. The two-stage crosslinking process invented by Finkelmann was applied to fabricate the modified polysiloxane side-chain LCE whose network contained mesogens and nonliquid crystal side chains linked to the polymer backbone, while the normal polysiloxane side-chain LCE did not contain nonliquid crystal side chains. Some salient properties were observed: The *T*_ni_ of the modified nematic LCE was significantly lowered than that of the normal nematic LCE by about 27 °C; compared to the thermo-actuation or photo-actuation of the normal nematic LCE or its graphene filled nanocomposite, the modified nematic LCE or its graphene filled nanocomposite demonstrated more rapid deformation speed, and the temperatures or luminance intensities to attain the same contraction ratios were obviously decreased. The temperature for the modified nematic LCE to attain the full axial contraction was lower than that for the normal nematic LCE by about 20 °C, and the luminance intensity for the modified nematic LCE nanocomposite to attain the full axial contraction was lower than that for the normal nematic LCE by about 2 × 10^5^ lux, which is about two times of the intensity of maximum natural sunlight; the maximum contraction ratio, which was about one-third, was not attenuated by the incorporation of nonliquid crystal chains into the network of the LCE matrix.

The research results indicates that the incorporation of nonliquid crystal chains into LCEs can become an effective way to improve the actuation properties of LCE materials, and hence promote the development of the application research of LCE materials.

## Conflicts of interest

The authors declare no competing financial interest.

## Supplementary Material
